# Structural characterization of two tetra­chlorido­zincate salts of 4-carb­oxy-1*H*-imidazol-3-ium: a salt hydrate and a co-crystal salt hydrate

**DOI:** 10.1107/S2056989017000317

**Published:** 2017-01-13

**Authors:** Sean J. Martens, David K Geiger

**Affiliations:** a Department of Chemistry, SUNY–College at Geneseo, Geneseo, New York 14454, USA

**Keywords:** crystal structure, co-crystal, hydrate, tetra­chlorido­zincate, imidazolium

## Abstract

Imidazole-containing compounds exhibit a myriad of pharmacological activities. Two tetra­chlorido­zincate salts of 4-carb­oxy-1*H*-imidazol-3-ium, ImHCO_2_H^+^, are reported. Bis(4-carb­oxy-1*H*-imidazol-3-ium) tetra­chlorido­zincate monohydrate, (C_4_H_5_N_2_O_2_)_2_[ZnCl_4_]·H_2_O, (I), crystallizes as a monohydrate salt, while bis­(4-carb­oxy-1*H*-imidazol-3-ium) tetra­chlorido­zincate bis­(1*H*-imidazol-3-ium-4-carboxyl­ato) monohydrate, (C_4_H_5_N_2_O_2_)_2_[ZnCl_4_]·2C_4_H_4_N_2_O_2_·H_2_O, (II), is a co-crystal salt with six residues: two ImHCO_2_H^+^ cations, two formula units of the zwitterionic 1*H*-imidazol-3-ium-4-carboxyl­ate, ImHCO_2_, one tetra­chlorido­zincate anion and one water mol­ecule disordered over two sites in a 0.60 (4):0.40 (4) ratio. The geometric parameters of the ImHCO_2_H^+^ and the ImHCO_2_ moieties are the same within the standard uncertainties of the measurements. Both compounds exhibit extensive hydrogen bonding, including involvement of the tetra­chlorido­zincate anion, resulting in inter­connected chains of anions joined by water mol­ecules.

## Abstract 2

Two tetra­chlorido­zincate salts of 4-carb­oxy-1*H*-imidazol-3-ium were structurally characterized. The first crystallizes with a water mol­ecule of hydration and the second with a water of hydration and two equivalents of the zwitterion 4-carb­oxy-1*H*-imidazole per salt formula unit.

## Chemical context   

Imidazole-containing compounds find use in numerous pharmaceuticals including fungicides, anti­viral agents, anti­arrhythmics, anti­histamines, and anthelmintics (Varala *et al.*, 2007 ▸; Horton *et al.*, 2003 ▸; López-Rodríguez *et al.*, 1999 ▸). Recent studies have shown that imidazole and benzimidazole derivatives exhibit pharmacological activity in histamine signaling (Tichenor *et al.*, 2015 ▸; Marson, 2011 ▸), and act as tau aggregation inhibitors for Alzheimer’s disease (Bulic *et al.*, 2013 ▸), and in the central nervous system (Robichaud *et al.*, 2011 ▸; Sheffler *et al.*, 2011 ▸). Further, derivatized imidazole-5-carb­oxy­lic acids have been shown to be angiotensin-converting enzyme (ACE) inhibitors (Jallapally *et al.*, 2015 ▸; Li *et al.*, 1998 ▸; Yanagisawa *et al.*, 1996 ▸).

As a result of the myriad binding modes available to imidazole ligands that bear carb­oxy­lic acid substituents, they have found use in the preparation of several metal organic frameworks, MOFs (Starosta & Leciejewicz, 2006 ▸; Yin *et al.*, 2009 ▸, 2012 ▸; Sun & Yang, 2007 ▸; Sun *et al.*, 2006 ▸). The synthesis and characterization of novel MOFs is an area of active research because of their potential use in such diverse areas as gas storage, catalysis, chemical sensors and mol­ecular separation (Dey *et al.*, 2014 ▸; Kreno *et al.*, 2012 ▸; Farha & Hupp, 2010 ▸). Neutral carb­oxy­imidazoles exist in their zwitterionic form and none of the reported compounds have the carb­oxy­imidazole ligand in the fully protonated form. However, there are examples of MOFs with anionic repeating units and imidazolium cations (Shao & Yu, 2014 ▸; Wang *et al.*, 2013 ▸).
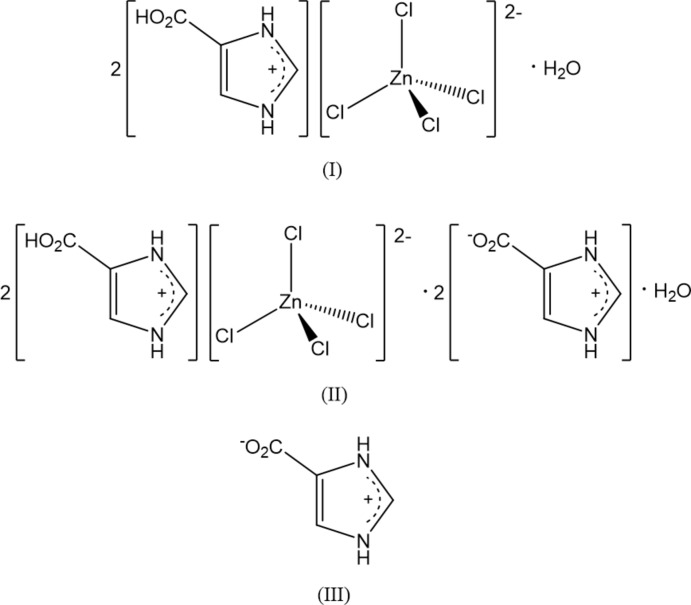


Although the structures of the zwitterionic 1*H*-imidazol-3-ium-4-carboxyl­ate, (III), (Cao *et al.*, 2012 ▸) and the corresponding 2-isopropyl (Du *et al.*, 2011 ▸) and 2-methyl (Guo, 2009 ▸) derivatives have been reported, to our knowledge, fully protonated imidazole­carb­oxy­lic acid species have not been structurally characterized. Compounds (I) and (II) possess the carb­oxy­imidazole in its fully protonated form and so contribute to the knowledge base of this class of compounds.

## Structural commentary   

**Figure 1:**
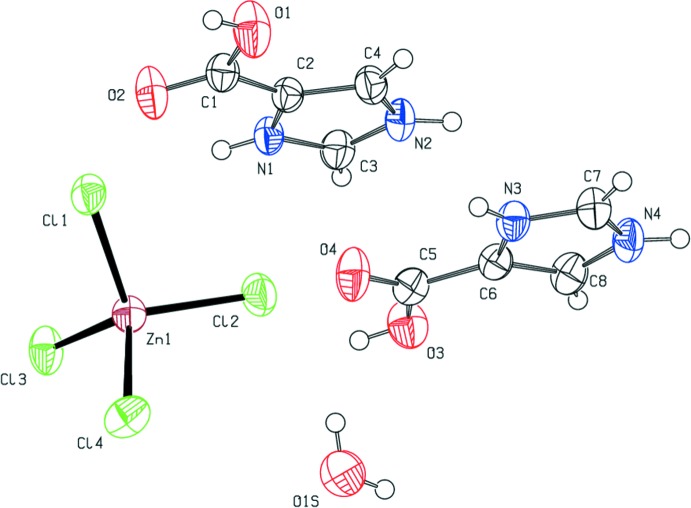
The mol­ecular structure of (I), showing the atom-labeling scheme. Non-hydrogen anisotropic displacement parameters are drawn at the 50% probability level.

Fig. 1 ▸ shows the atom-labeling scheme employed for (I). The asymmetric unit consists of two 4-carb­oxy-1*H*-imidazol-3-ium cations (ImHCO_2_H^+^), one tetra­chlorido­zincate anion, and one water mol­ecule. Thus, compound (I) is classified as a salt solvate (Grothe *et al.*, 2016 ▸) with four residues.

**Figure 2:**
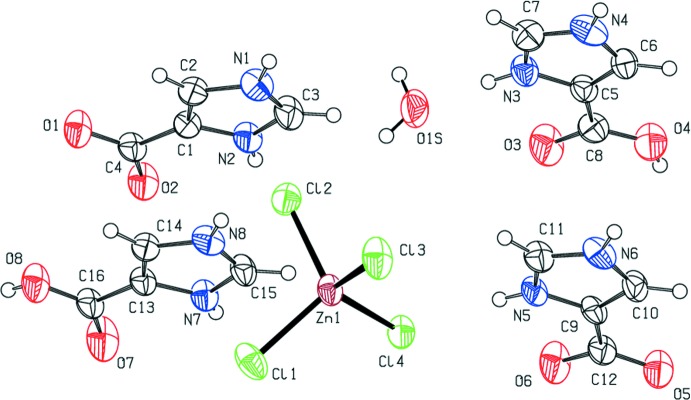
The mol­ecular structure of (II), showing the atom-labeling scheme. Non-hydrogen anisotropic displacement parameters are drawn at the 50% probability level.

Compound (II) is an example of a rare co-crystal salt solvate with six residues (Grothe *et al.*, 2016 ▸). The asymmetric unit consists of two ImHCO_2_H^+^ cations, one tetra­chlorido­zincate anion, two 1*H*-imidazol-3-ium-4-carboxylate zwitterions (ImHCO_2_), and one water mol­ecule. The atom-labeling scheme employed is shown in Fig. 2 ▸.

The geometric parameters determined for the tetra­chlorido­zincate anions in (I) and (II) are found in Tables 1 ▸ and 2 ▸, respectively. The average Zn—Cl bond length is 2.273 (3) and 2.272 (15) Å, respectively, for (I) and (II), which are within the range 2.2409 (3)–2.3085 (7) Å found in other examples of tetra­chlorido­zincate salts (Govindan *et al.*, 2014*a* ▸,*b* ▸; Leesakul *et al.*, 2012 ▸; Goh *et al.*, 2012 ▸; Kefi *et al.*, 2011 ▸). The same example structures exhibit Cl—Zn—Cl angles in the range 102.256 (10) to 112.72 (3)°. The average angles found in (I) and (II) are 109 (2)° and 109 (3)°, respectively, and the individual values exhibit comparable ranges (Tables 1 ▸ and 2 ▸).

There are no noteworthy differences in the C—C and C—N bond lengths between the ImHCO_2_H^+^ cations and ImHCO_2_ zwitterions found in (I) and (II). The N—C′ bond length, where C′ is the carbon atom bonded to both nitro­gen atoms in a given ring (formally, the 2 position in the ring), is consistently shorter than the N—C′′ bond length, where C′′ represents the carbon in the formal 4 or 5 position in the ring, for all of the ImHCO_2_H^+^ and ImHCO_2_ residues. This observation is consistent with other reported imidazoles and imidazolium salts (*e.g*., Mohamed *et al.*, 2014 ▸; Trifa *et al.*, 2013 ▸; Chérif *et al.*, 2013 ▸; Yu, 2012 ▸; Zhu, 2012 ▸).

The carb­oxy and carboxyl­ate groups are tilted slightly from the imidazole plane in all cases. The N—C—C—O torsion angles are reported in Tables 1 ▸ and 2 ▸. In both (I) and (II), the carb­oxy and carboxyl­ate groups are unsymmetrical. For the carb­oxy groups, the C—OH bond is longer than the C=O bond. These observations are consistent with the geometric parameters found in similar imidazole­carb­oxy­lic acids (Cao *et al.*, 2012 ▸; Du *et al.*, 2011 ▸; Guo, 2009 ▸). The observed O—C—O bond angles of the fully protonated form in (I) and (II) and the zwitterionic form in (II) are the same within the standard uncertainties of the refinement.

## Supra­molecular features   

**Figure 3:**
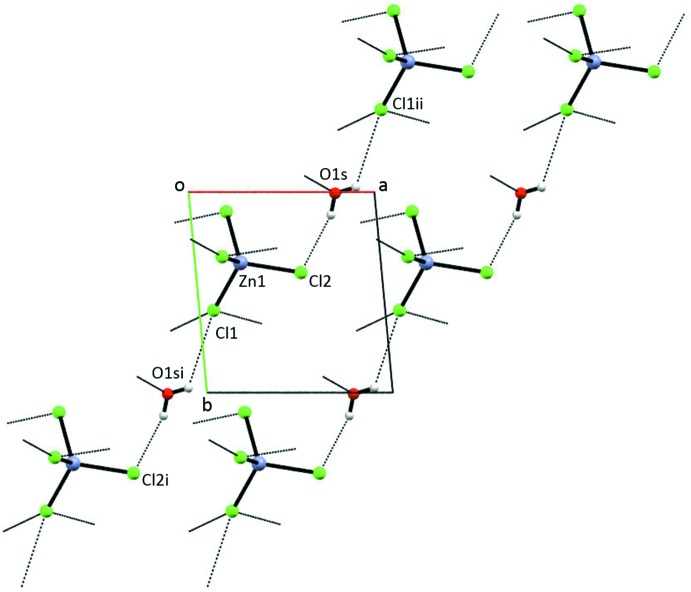
Partial packing diagram of (I), showing the water–tetra­chlorido­zincate chains. Only the tetra­chlorido­zincate anion and the water of hydration are shown.

**Figure 4:**
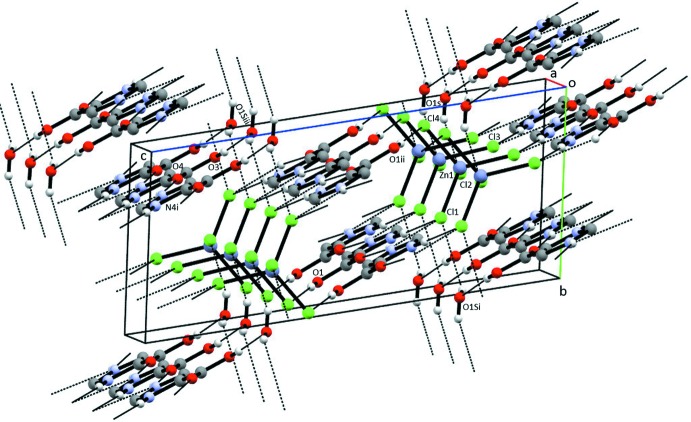
Packing diagram of (I), showing the hydrogen-bonding scheme. Only H atoms involved in the inter­actions are shown.

An extensive hydrogen-bonding network in (I) involving the tetra­chlorido­zincate anion and the water of hydration results in chains parallel to [2
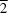
0], as seen in Fig. 3 ▸ and Table 3 ▸. Additional Cl⋯H—O—H⋯Cl inter­actions along [100] join the chains (Fig. 4 ▸). N—H⋯O(water), N—H⋯Cl, and O—H⋯O hydrogen bonds incorporate the ImHCO_2_H^+^ cations into the three-dimensional extended structure. Using graph-set analysis to describe the hydrogen bonding (Etter *et al.*, 1990 ▸), an 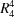
(20) ring is observed with four oxygen acceptors, two oxygen donors and two nitro­gen donors. One oxygen donor, two oxygen acceptor rings, 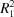
(4), involving a carb­oxy group are also present.

**Figure 5:**
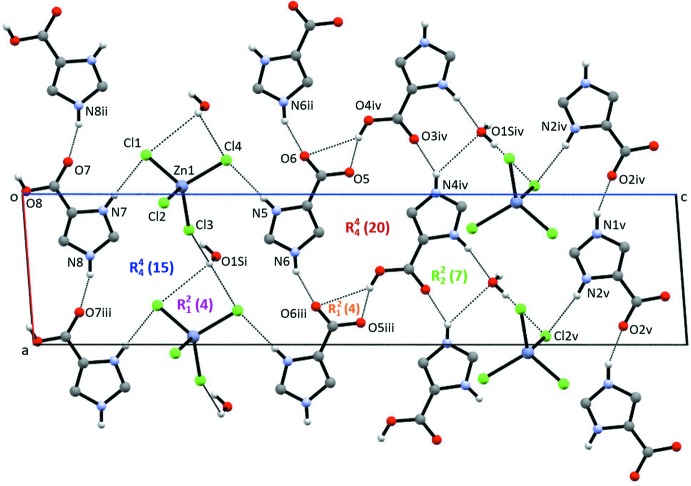
A view of the hydrogen bonding in (II), showing the ring motifs. Only H atoms involved in the inter­actions are shown. Only the major contributor to the disorder model of the water mol­ecule is shown.

Figs. 5 ▸ and 6 ▸ show two views of the crystal packing observed in (II). Hydrogen-bonding parameters are found in Table 4 ▸. As seen in Fig. 5 ▸, there are several hydrogen-bonding ring motifs that are common to (I) and (II). An 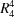
(20) ring is observed with four oxygen acceptors, two oxygen donors and two nitro­gen donors, and there is a one oxygen donor, two oxygen acceptor ring, 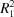
(4), involving a carb­oxy group. 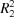
(7) rings involving two nitro­gen donors and two oxygen acceptors are also observed. There are two rings containing chlorine acceptor atoms: an 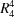
(15) system with one oxygen donor, three nitro­gen donors, one oxygen acceptor and three chlorine acceptors; and an 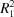
(4) ring with a single oxygen donor and two chlorine acceptors. Similarly to (I), chains of hydrogen-bonded tetra­chlorido­zincate anions and water mol­ecules of hydration are found parallel to [2
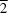
0].

In (I), a weak π–π inter­action between ImHCO_2_H^+^ cations related by a crystallographically imposed center of symmetry is observed with a centroid-to-centroid distance of 3.5781 (15) Å and an inter­planar distance of 3.4406 (9) Å, corresponding to 0.983 Å slippage (Spek, 2009 ▸). Two independent weak π–π inter­actions between ImHCO_2_H^+^ cations and ImHCO_2_ zwitterions are observed in (II). The principal one involves the rings containing N1 and N7 with a centroid-to-centroid distance of 3.5871 (3) Å, an inter­planar distance of 3.3591 (18) Å and a dihedral angle of 2.6 (2)° between rings (Spek, 2009 ▸). The weaker π–π inter­action involves the rings containing N3 and N5 and has a centroid-to-centroid distance of 3.740 (3) Å, an inter­planar distance of 3.3140 (17) Å and a dihedral angle of 1.2 (2) Å between planes.

**Figure 7:**
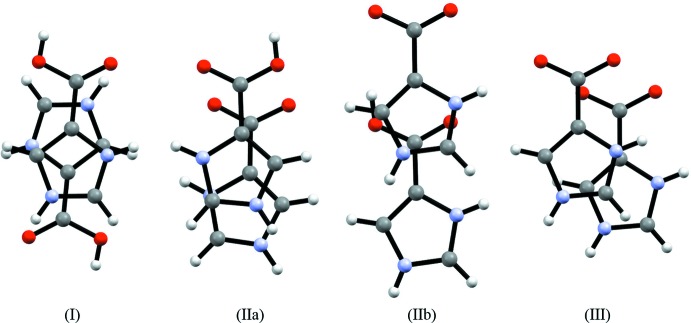
Projections of π-stacked mol­ecules. The mol­ecules are related by the symmetry transformations (I) −*x* + 1, −*y* + 1, −*z* + 1; (IIa) *x*, *y*, *z*; (IIb) *x* + 1, *y* − 1,*z*; (III) *x*, *y*, *z* + 1.

Fig. 7 ▸ shows representations of the observed π stacking in which members of inter­acting pairs of mol­ecules are projected into the same plane. A π–π inter­action is also observed in the solid-state structure of the ImHCO_2_ zwitterion, labeled (III) (Cao *et al.*, 2012 ▸). In (III), the centroid–centroid distance is longer [3.674 (4) Å] than that observed between the fully protonated form in (I) and the principal inter­action between the zwitterion and the protonated form in (II). In all of the pairs except in (I), the members of the pairs are arranged in a head-to-head configuration.

## Database survey   

The structure of 1-*H*-imidazol-3-ium-4-carboxyl­ate has been reported (Cao *et al.*, 2012 ▸) and the structures of the 2-methyl and 2-isopropyl derivatives of the zwitterion 5-carb­oxy-1*H*-3-ium-4-carboxyl­ate monohydrate have been reported (Guo, 2009 ▸; Du *et al.*, 2011 ▸). Several polymeric compounds with bridging 1*H*-imidazole-4-carboxyl­ato ligands have been reported, including one with Ca^II^ (Starosta & Leciejewicz, 2006 ▸) and two with Cd^II^ (Yin *et al.*, 2009 ▸, 2012 ▸). The structures of monomeric compounds with 1*H*-imidazole-4-carboxyl­ato-*κ* ^2^ *N,O* ligands and Mg^II^ (Gryz *et al.*, 2007 ▸), Mn^II^ (Xiong *et al.*, 2013 ▸), Co^II^ (Chen, 2012 ▸; Artetxe *et al.*, 2013 ▸), Ni^II^ (Zheng *et al.*, 2011 ▸), Cu^II^ (Reinoso *et al.*, 2015 ▸), and Zn^II^ (Gryz *et al.*, 2007 ▸; He, 2006 ▸; Shuai *et al.*, 2011 ▸) have been determined. Tetra­nuclear Mn^II^ complexes with 1*H*-imidazole-4-carboxyl­ato-*κ* ^2^ *N,O* and the structurally similar 4-imidazole­acetate ligand have also been characterized (Boskovic *et al.*, 2000 ▸). The structures of numerous imidazolium salts are known (*e.g.*, Mohamed *et al.*,, 2014 ▸; Trifa *et al.*, 2013 ▸; Chérif *et al.*, 2013 ▸; Yu, 2012 ▸; Zhu, 2012 ▸; Ishida & Kashino, 2001 ▸; Gili *et al.*, 2000 ▸; Pavan Kumar & Kumara Swamy, 2005 ▸; Hashizume *et al.*, 2001 ▸; Moreno-Fuquen *et al.*, 2009*a* ▸,*b* ▸, 2011 ▸; Zhang *et al.*, 2011 ▸; Sun *et al.*, 2002 ▸; Fukunaga & Ishida, 2003 ▸). There are many examples of reported structures of tetra­chlorido­zincate salts (*e.g*., Govindan *et al.*, 2014*a* ▸,*b* ▸; Leesakul *et al.*, 2012 ▸; Goh *et al.*, 2012 ▸; Kefi *et al.*, 2011 ▸).

## Synthesis and crystallization   

Compounds (I) and (II) were obtained during the attempted syntheses of Zn^II^ coordination polymers. (I) was obtained by dissolving 113 mg (0.829 mmol) ZnCl_2_ and 194 mg (1.73 mmol) 1*H*-imidazole-4-carb­oxy­lic acid in ethanol. Six drops of 6 *M* HCl were added and the mixture was heated to reflux with stirring. The warm solution was filtered and the filtrate was allowed to cool. After a few days, crystalline clumps of the product were obtained. ^1^H NMR (400 MHz, dmso-*d*_6_, p.p.m.): 7.97 (*s*, 2H), 8.51 (*s*, 2H). ^13^C NMR (100 MHz, dmso-*d*_6_, p.p.m.): 126.1, 127.4, 137.7, 161.3. A crystal cut from a larger mass of crystals was used for X-ray analysis.

Compound (II) was prepared similarly to (I) except that methanol was the solvent and no HCl was added to the reaction mixture. Single crystals for X-ray analysis were obtained by slow evaporation of a methanol solution.^1^H NMR (400 MHz, dmso-*d*_6_, p.p.m.): 8.15 (*s*, 4H), 8.95 (*s*, 4H). ^13^C NMR (100 MHz, dmso-*d*_6_, p.p.m.): 125.4, 126.1, 137.6, 160.3.

## Refinement   

Crystal data, data collection and structure refinement details are summarized in Table 5 ▸. For (I), data completeness was 97.9% and for (II) it was 95.2%. For both (I) and (II), all hydrogen atoms were located in difference Fourier maps. The hydrogen atoms bonded to carbon were refined using a riding model with a C—H distance of 0.95 Å and hydrogen-atom isotropic displacement parameters were set using the approximation *U*_iso_(H) = 1.2*U*_eq_(C). The O—H and N—H distances were restrained to 0.84 and 0.88 Å, respectively. The isotropic displacement parameters of the hydrogen atoms bonded to nitro­gen were set using the approximation *U*_iso_(H) = 1.2*U*_eq_(N). In (I), isotropic displacement parameters of the hydrogen atoms bonded to oxygen were refined freely, but for (II) they were set using the approximation *U*_iso_(H) = 1.5*U*_eq_(O). For (II), the water mol­ecule is disordered over two positions. In addition to the aforementioned distance restraint, an H—O—H angle restraint of 105° was employed. The occupancies refined to 0.60 (4):0.40 (4).

## Supplementary Material

Crystal structure: contains datablock(s) global, I, II. DOI: 10.1107/S2056989017000317/pj2040sup1.cif

Structure factors: contains datablock(s) I. DOI: 10.1107/S2056989017000317/pj2040Isup2.hkl

Supporting information file. DOI: 10.1107/S2056989017000317/pj2040Isup4.mol

Structure factors: contains datablock(s) II. DOI: 10.1107/S2056989017000317/pj2040IIsup3.hkl

Supporting information file. DOI: 10.1107/S2056989017000317/pj2040IIsup5.mol

CCDC references: 1526096, 1526095

Additional supporting information:  crystallographic information; 3D view; checkCIF report

## Figures and Tables

**Figure 6:**
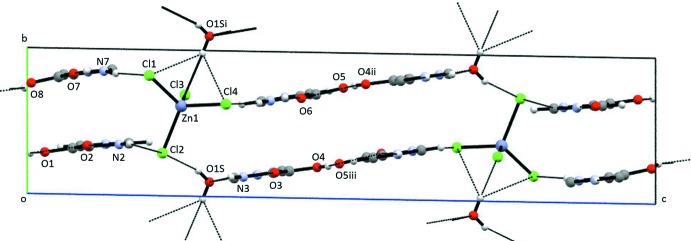
A second view of the hydrogen bonding in (II). Only H atoms involved in the hydrogen bonds are shown. Only the major contributor to the disorder model of the water mol­ecule is shown.

**Table 1:** Selected geometric parameters (Å, °) for (I)

Zn1—Cl2	2.2690 (7)	O1—C1	1.313 (2)
Zn1—Cl3	2.2704 (6)	O2—C1	1.200 (2)
Zn1—Cl4	2.2737 (6)	O3—C5	1.305 (2)
Zn1—Cl1	2.2794 (6)	O4—C5	1.201 (3)
			
Cl2—Zn1—Cl3	107.93 (2)	Cl3—Zn1—Cl1	112.84 (2)
Cl2—Zn1—Cl4	111.11 (2)	Cl4—Zn1—Cl1	107.92 (2)
Cl3—Zn1—Cl4	109.21 (3)	O2—C1—O1	124.9 (2)
Cl2—Zn1—Cl1	107.85 (2)	O4—C5—O3	125.4 (2)
			
O2—C1—C2—N1	4.6 (3)	O4—C5—C6—N3	−1.8 (3)

**Table 2:** Selected geometric parameters (Å, °) for (II)

Zn1—Cl3	2.2577 (12)	O3—C8	1.220 (5)
Zn1—Cl4	2.2589 (11)	O4—C8	1.277 (5)
Zn1—Cl1	2.2758 (12)	O5—C12	1.274 (5)
Zn1—Cl2	2.2948 (12)	O6—C12	1.222 (5)
O1—C4	1.271 (4)	O7—C16	1.224 (5)
O2—C4	1.229 (4)	O8—C16	1.267 (5)
			
Cl3—Zn1—Cl4	111.21 (4)	Cl1—Zn1—Cl2	108.62 (5)
Cl3—Zn1—Cl1	112.42 (5)	O2—C4—O1	126.3 (4)
Cl4—Zn1—Cl1	109.31 (5)	O3—C8—O4	125.6 (4)
Cl3—Zn1—Cl2	104.23 (5)	O6—C12—O5	126.3 (4)
Cl4—Zn1—Cl2	110.95 (4)	O7—C16—O8	126.3 (4)
			
N2—C1—C4—O2	5.7 (6)	N5—C9—C12—O6	3.2 (6)
N3—C5—C8—O3	−2.3 (6)	N7—C13—C16—O7	6.3 (6)

**Table 3:** Hydrogen-bond geometry (Å, °) for (I)

*D*—H⋯*A*	*D*—H	H⋯*A*	*D*⋯*A*	*D*—H⋯*A*
O1*S*—H1*SA*⋯Cl2	0.83 (2)	2.42 (2)	3.192 (3)	155 (4)
O1*S*—H1*SB*⋯Cl4^i^	0.81 (2)	2.94 (4)	3.392 (2)	118 (3)
O1*S*—H1*SB*⋯Cl1^ii^	0.81 (2)	3.05 (4)	3.540 (3)	122 (4)
O1—H1*A*⋯Cl4^iii^	0.83 (2)	2.25 (2)	3.0723 (19)	171 (3)
O3—H3*A*⋯O1*S* ^iv^	0.83 (2)	1.77 (2)	2.576 (3)	163 (3)
N1—H1*N*⋯Cl1	0.84 (2)	2.37 (2)	3.2105 (17)	178 (2)
N2—H2*N*⋯Cl1^i^	0.86 (2)	2.85 (2)	3.3787 (19)	122 (2)
N2—H2*N*⋯O2^i^	0.86 (2)	1.99 (2)	2.745 (2)	146 (2)
N3—H3*N*⋯Cl3^v^	0.83 (2)	2.37 (2)	3.1941 (18)	177 (2)
N4—H4*N*⋯Cl3^vi^	0.86 (2)	2.83 (2)	3.3586 (19)	121 (2)
N4—H4*N*⋯O4^i^	0.86 (2)	2.00 (2)	2.753 (2)	146 (2)

Symmetry codes: (i) 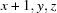; (ii) 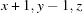; (iii) 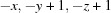; (iv) 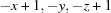; (v) 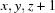; (vi) 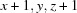.

**Table 4:** Hydrogen-bond geometry (Å, °) for (II)

*D*—H⋯*A*	*D*—H	H⋯*A*	*D*⋯*A*	*D*—H⋯*A*
O4—H4*A*⋯O5^i^	0.87 (2)	1.63 (2)	2.476 (4)	163 (5)
O4—H4*A*⋯O6^i^	0.87 (2)	2.52 (4)	3.205 (4)	136 (4)
O8—H8*A*⋯O1^ii^	0.85 (2)	1.65 (2)	2.484 (4)	166 (5)
O8—H8*A*⋯O2^ii^	0.85 (2)	2.63 (4)	3.162 (4)	122 (4)
O1*S*—H1*A*⋯Cl2	0.85 (2)	2.52 (5)	3.330 (9)	159 (10)
O1*S*—H1*B*⋯Cl1^iii^	0.84 (2)	2.97 (9)	3.546 (15)	128 (10)
O1*S*—H1*B*⋯Cl4^iii^	0.84 (2)	2.93 (7)	3.499 (11)	127 (8)
O2*S*—H2*A*⋯Cl2	0.85 (2)	2.61 (12)	3.382 (17)	150.20
O2*S*—H2*B*⋯Cl1^iii^	0.85 (2)	2.36 (10)	3.13 (2)	150 (17)
N1—H1*N*⋯O2^iv^	0.88 (2)	1.86 (2)	2.712 (4)	160 (4)
N2—H2*N*⋯Cl2	0.87 (2)	2.44 (2)	3.297 (3)	167 (4)
N3—H3*N*⋯O1*S*	0.87 (2)	2.01 (2)	2.860 (10)	168 (4)
N3—H3*N*⋯O2*S*	0.87 (2)	1.89 (2)	2.739 (12)	165 (4)
N4—H4*N*⋯O3^iv^	0.85 (2)	1.92 (3)	2.677 (5)	148 (4)
N5—H5*N*⋯Cl4	0.86 (2)	2.39 (2)	3.247 (3)	173 (4)
N6—H6*N*⋯O6^iv^	0.87 (2)	1.85 (3)	2.661 (4)	156 (4)
N7—H7*N*⋯Cl1	0.89 (2)	2.32 (2)	3.205 (3)	175 (4)
N8—H8*N*⋯O7^iv^	0.87 (2)	1.78 (2)	2.628 (4)	164 (4)

Symmetry codes: (i) 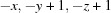; (ii) 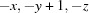; (iii) 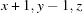; (iv) 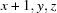.

**Table 5:** Experimental details

	(I)	(II)
Crystal data		
Chemical formula	(C_4_H_5_N_2_O_2_)_2_[ZnCl_4_]·H_2_O	(C_4_H_4_N_2_O_2_)_2_[ZnCl_4_]·2C_4_H_5_N_2_O_2_H_2_O
*M*_r_	451.39	675.57
Crystal system, space group	Triclinic, *P* 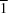	Triclinic, *P* 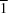
Temperature (K)	200	200
*a*, *b*, *c* (Å)	6.9094 (10), 7.5828 (12), 16.468 (3)	6.9369 (19), 6.9624 (15), 28.483 (8)
α, β, γ (°)	79.455 (4), 84.489 (4), 83.833 (4)	89.524 (9), 85.622 (9), 71.202 (8)
*V* (Å^3^)	840.7 (2)	1298.3 (6)
*Z*	2	2
Radiation type	Mo *K*α	Mo *K*α
μ (mm^−1^)	2.12	1.42
Crystal size (mm)	0.60 × 0.50 × 0.20	0.50 × 0.25 × 0.20

Data collection		
Diffractometer	Bruker SMART X2S benchtop	Bruker SMART X2S benchtop
Absorption correction	Multi-scan (*SADABS*; Bruker, 2013 ▸)	Multi-scan (*SADABS*; Bruker, 2013 ▸)
*T*_min_, *T*_max_	0.41, 0.68	0.50, 0.76
No. of measured, independent and observed [*I* > 2σ(*I*)] reflections	10429, 3375, 2995	10560, 4855, 3619
*R*_int_	0.032	0.042
(sin θ/λ)_max_ (Å^−1^)	0.625	0.616

Refinement		
*R*[*F* ^2^ > 2σ(*F* ^2^)], *wR*(*F* ^2^), *S*	0.024, 0.064, 1.03	0.050, 0.135, 1.03
No. of reflections	3375	4855
No. of parameters	227	395
No. of restraints	8	16
H-atom treatment	H atoms treated by a mixture of independent and constrained refinement	H atoms treated by a mixture of independent and constrained refinement
Δρ_max_, Δρ_min_ (e Å^−3^)	0.53, −0.28	0.63, −0.71

Computer programs: *APEX2* and *SAINT* (Bruker, 2013 ▸), *SHELXS97* (Sheldrick, 2008 ▸), *SHELXL2014* (Sheldrick, 2015 ▸), *PLATON* (Spek, 2009 ▸), *Mercury* (Macrae *et al.*, 2006 ▸) and *publCIF* (Westrip, 2010 ▸).

## supplementary crystallographic information

### (I) Bis(4-carboxy-1H-imidazol-3-ium) tetrachloridozincate monohydrate Crystal data

**Table:**

(C_4_H_5_N_2_O_2_)_2_[ZnCl_4_]·H_2_O	*Z* = 2
*M**_r_* = 451.39	*F*(000) = 452
Triclinic, *P*1	*D*_x_ = 1.783 Mg m^−^^3^
*a* = 6.9094 (10) Å	Mo *K*α radiation, λ = 0.71073 Å
*b* = 7.5828 (12) Å	Cell parameters from 6894 reflections
*c* = 16.468 (3) Å	θ = 2.5–27.5°
α = 79.455 (4)°	µ = 2.12 mm^−^^1^
β = 84.489 (4)°	*T* = 200 K
γ = 83.833 (4)°	Plate, clear colourless
*V* = 840.7 (2) Å^3^	0.60 × 0.50 × 0.20 mm

### (I) Bis(4-carboxy-1H-imidazol-3-ium) tetrachloridozincate monohydrate Data collection

**Table:**

Bruker SMART X2S benchtop diffractometer	3375 independent reflections
Radiation source: sealed microfocus tube	2995 reflections with *I* > 2σ(*I*)
Doubly curved silicon crystal monochromator	*R*_int_ = 0.032
Detector resolution: 8.3330 pixels mm^-1^	θ_max_ = 26.4°, θ_min_ = 2.5°
ω scans	*h* = −8→8
Absorption correction: multi-scan (SADABS; Bruker, 2013)	*k* = −9→9
*T*_min_ = 0.41, *T*_max_ = 0.68	*l* = −20→19
10429 measured reflections	

### (I) Bis(4-carboxy-1H-imidazol-3-ium) tetrachloridozincate monohydrate Refinement

**Table:**

Refinement on *F*^2^	Primary atom site location: structure-invariant direct methods
Least-squares matrix: full	Secondary atom site location: difference Fourier map
*R*[*F*^2^ > 2σ(*F*^2^)] = 0.024	Hydrogen site location: mixed
*wR*(*F*^2^) = 0.064	H atoms treated by a mixture of independent and constrained refinement
*S* = 1.03	*w* = 1/[σ^2^(*F*_o_^2^) + (0.0292*P*)^2^ + 0.2531*P*] where *P* = (*F*_o_^2^ + 2*F*_c_^2^)/3
3375 reflections	(Δ/σ)_max_ = 0.001
227 parameters	Δρ_max_ = 0.53 e Å^−^^3^
8 restraints	Δρ_min_ = −0.28 e Å^−^^3^

### (I) Bis(4-carboxy-1H-imidazol-3-ium) tetrachloridozincate monohydrate Special details

**Table:**

Geometry. All esds (except the esd in the dihedral angle between two l.s. planes) are estimated using the full covariance matrix. The cell esds are taken into account individually in the estimation of esds in distances, angles and torsion angles; correlations between esds in cell parameters are only used when they are defined by crystal symmetry. An approximate (isotropic) treatment of cell esds is used for estimating esds involving l.s. planes.

### (I) Bis(4-carboxy-1H-imidazol-3-ium) tetrachloridozincate monohydrate Fractional atomic coordinates and isotropic or equivalent isotropic displacement parameters (Å^2^)

**Table:**

	*x*	*y*	*z*	*U*_iso_*/*U*_eq_	
Zn1	0.24426 (3)	0.35245 (3)	0.23892 (2)	0.03225 (8)	
Cl1	0.07702 (7)	0.58951 (7)	0.29003 (3)	0.04037 (13)	
Cl2	0.56514 (8)	0.40109 (8)	0.22143 (4)	0.04596 (14)	
Cl3	0.14855 (8)	0.32216 (8)	0.11448 (3)	0.04391 (14)	
Cl4	0.19391 (9)	0.09768 (8)	0.33224 (3)	0.04754 (14)	
O1S	0.7881 (4)	0.0065 (3)	0.25272 (16)	0.0744 (6)	
H1SA	0.755 (6)	0.114 (3)	0.256 (2)	0.113 (16)*	
H1SB	0.902 (3)	−0.023 (6)	0.241 (3)	0.112 (17)*	
O1	0.2088 (3)	0.8341 (3)	0.57654 (11)	0.0583 (5)	
H1A	0.096 (3)	0.846 (4)	0.5976 (19)	0.080 (11)*	
O2	0.0547 (2)	0.7334 (2)	0.48378 (10)	0.0499 (4)	
O3	0.4700 (2)	0.1089 (2)	0.82247 (10)	0.0488 (4)	
H3A	0.371 (3)	0.082 (4)	0.8047 (18)	0.068 (9)*	
O4	0.2491 (2)	0.1786 (3)	0.92209 (10)	0.0509 (4)	
N1	0.4180 (2)	0.6761 (2)	0.39214 (10)	0.0328 (4)	
H1N	0.328 (3)	0.656 (3)	0.3653 (13)	0.039*	
N2	0.7041 (3)	0.6977 (3)	0.42572 (12)	0.0398 (4)	
H2N	0.830 (2)	0.694 (3)	0.4249 (15)	0.048*	
N3	0.5549 (2)	0.2595 (2)	1.00903 (11)	0.0354 (4)	
H3N	0.448 (3)	0.280 (3)	1.0351 (14)	0.042*	
N4	0.8615 (3)	0.2308 (3)	0.97687 (12)	0.0414 (4)	
H4N	0.985 (2)	0.234 (3)	0.9774 (16)	0.05*	
C1	0.2014 (3)	0.7670 (3)	0.50896 (12)	0.0340 (4)	
C2	0.3956 (3)	0.7354 (3)	0.46709 (12)	0.0304 (4)	
C3	0.6053 (3)	0.6554 (3)	0.36821 (13)	0.0374 (5)	
H3	0.6597	0.6168	0.3185	0.045*	
C4	0.5780 (3)	0.7487 (3)	0.48777 (13)	0.0365 (4)	
H4	0.6113	0.7864	0.5362	0.044*	
C5	0.4145 (3)	0.1639 (3)	0.89239 (12)	0.0352 (4)	
C6	0.5806 (3)	0.2008 (3)	0.93398 (12)	0.0320 (4)	
C7	0.7275 (3)	0.2773 (3)	1.03332 (14)	0.0404 (5)	
H7	0.7507	0.3166	1.0828	0.048*	
C8	0.7758 (3)	0.1833 (3)	0.91435 (13)	0.0395 (5)	
H8	0.8402	0.1452	0.8661	0.047*	

### (I) Bis(4-carboxy-1H-imidazol-3-ium) tetrachloridozincate monohydrate Atomic displacement parameters (Å^2^)

**Table:**

	*U*^11^	*U*^22^	*U*^33^	*U*^12^	*U*^13^	*U*^23^
Zn1	0.03082 (14)	0.03910 (14)	0.02828 (13)	−0.00235 (10)	−0.00091 (9)	−0.01089 (10)
Cl1	0.0302 (3)	0.0506 (3)	0.0444 (3)	0.0027 (2)	−0.0016 (2)	−0.0234 (2)
Cl2	0.0303 (3)	0.0544 (3)	0.0579 (3)	−0.0051 (2)	−0.0010 (2)	−0.0226 (3)
Cl3	0.0329 (3)	0.0699 (4)	0.0337 (3)	−0.0025 (2)	−0.0048 (2)	−0.0219 (2)
Cl4	0.0491 (3)	0.0446 (3)	0.0421 (3)	0.0028 (2)	0.0056 (2)	0.0016 (2)
O1S	0.0736 (16)	0.0632 (14)	0.0966 (17)	0.0020 (12)	−0.0382 (14)	−0.0293 (12)
O1	0.0376 (10)	0.0984 (14)	0.0484 (10)	−0.0049 (9)	−0.0019 (8)	−0.0392 (10)
O2	0.0282 (8)	0.0812 (12)	0.0462 (9)	−0.0099 (8)	−0.0035 (7)	−0.0234 (8)
O3	0.0444 (10)	0.0668 (11)	0.0403 (9)	−0.0072 (8)	−0.0005 (7)	−0.0231 (8)
O4	0.0276 (8)	0.0815 (12)	0.0447 (9)	−0.0031 (8)	−0.0002 (7)	−0.0164 (8)
N1	0.0264 (9)	0.0406 (9)	0.0332 (9)	−0.0024 (7)	−0.0075 (7)	−0.0089 (7)
N2	0.0227 (9)	0.0491 (10)	0.0480 (10)	−0.0037 (8)	−0.0042 (8)	−0.0081 (8)
N3	0.0266 (9)	0.0453 (10)	0.0340 (9)	−0.0015 (8)	0.0024 (7)	−0.0098 (8)
N4	0.0235 (9)	0.0515 (11)	0.0478 (11)	−0.0049 (8)	−0.0014 (8)	−0.0046 (9)
C1	0.0316 (11)	0.0408 (11)	0.0299 (10)	−0.0025 (9)	−0.0045 (8)	−0.0064 (8)
C2	0.0286 (10)	0.0335 (10)	0.0297 (9)	−0.0016 (8)	−0.0055 (8)	−0.0060 (8)
C3	0.0308 (11)	0.0429 (11)	0.0383 (11)	−0.0020 (9)	−0.0016 (9)	−0.0080 (9)
C4	0.0323 (11)	0.0411 (11)	0.0381 (11)	−0.0051 (9)	−0.0085 (9)	−0.0082 (9)
C5	0.0343 (12)	0.0373 (11)	0.0324 (10)	−0.0011 (9)	−0.0016 (8)	−0.0034 (8)
C6	0.0295 (11)	0.0351 (10)	0.0298 (9)	−0.0017 (8)	0.0008 (8)	−0.0043 (8)
C7	0.0330 (12)	0.0484 (12)	0.0410 (11)	−0.0048 (9)	−0.0045 (9)	−0.0094 (10)
C8	0.0310 (11)	0.0479 (12)	0.0369 (11)	−0.0013 (9)	0.0063 (9)	−0.0062 (9)

### (I) Bis(4-carboxy-1H-imidazol-3-ium) tetrachloridozincate monohydrate Geometric parameters (Å, º)

**Table:**

Zn1—Cl2	2.2690 (7)	N2—C4	1.362 (3)
Zn1—Cl3	2.2704 (6)	N2—H2N	0.864 (16)
Zn1—Cl4	2.2737 (6)	N3—C7	1.321 (3)
Zn1—Cl1	2.2794 (6)	N3—C6	1.378 (2)
O1S—H1SA	0.829 (19)	N3—H3N	0.830 (16)
O1S—H1SB	0.806 (18)	N4—C7	1.316 (3)
O1—C1	1.313 (2)	N4—C8	1.356 (3)
O1—H1A	0.828 (18)	N4—H4N	0.858 (16)
O2—C1	1.200 (2)	C1—C2	1.465 (3)
O3—C5	1.305 (2)	C2—C4	1.355 (3)
O3—H3A	0.827 (17)	C3—H3	0.95
O4—C5	1.201 (3)	C4—H4	0.95
N1—C3	1.317 (3)	C5—C6	1.468 (3)
N1—C2	1.379 (2)	C6—C8	1.354 (3)
N1—H1N	0.838 (16)	C7—H7	0.95
N2—C3	1.323 (3)	C8—H8	0.95
			
Cl2—Zn1—Cl3	107.93 (2)	O1—C1—C2	112.04 (17)
Cl2—Zn1—Cl4	111.11 (2)	C4—C2—N1	106.33 (17)
Cl3—Zn1—Cl4	109.21 (3)	C4—C2—C1	132.70 (18)
Cl2—Zn1—Cl1	107.85 (2)	N1—C2—C1	120.95 (17)
Cl3—Zn1—Cl1	112.84 (2)	N1—C3—N2	107.86 (18)
Cl4—Zn1—Cl1	107.92 (2)	N1—C3—H3	126.1
H1SA—O1S—H1SB	119 (4)	N2—C3—H3	126.1
C1—O1—H1A	107 (2)	C2—C4—N2	106.69 (18)
C5—O3—H3A	107 (2)	C2—C4—H4	126.7
C3—N1—C2	109.33 (16)	N2—C4—H4	126.7
C3—N1—H1N	124.5 (16)	O4—C5—O3	125.4 (2)
C2—N1—H1N	126.2 (16)	O4—C5—C6	122.53 (18)
C3—N2—C4	109.79 (17)	O3—C5—C6	112.00 (18)
C3—N2—H2N	125.9 (17)	C8—C6—N3	106.23 (18)
C4—N2—H2N	124.3 (17)	C8—C6—C5	132.12 (18)
C7—N3—C6	109.06 (17)	N3—C6—C5	121.61 (17)
C7—N3—H3N	125.3 (17)	N4—C7—N3	107.89 (19)
C6—N3—H3N	125.6 (17)	N4—C7—H7	126.1
C7—N4—C8	110.01 (18)	N3—C7—H7	126.1
C7—N4—H4N	126.0 (17)	C6—C8—N4	106.81 (18)
C8—N4—H4N	123.9 (17)	C6—C8—H8	126.6
O2—C1—O1	124.9 (2)	N4—C8—H8	126.6
O2—C1—C2	123.11 (18)		
			
C3—N1—C2—C4	−0.3 (2)	C7—N3—C6—C8	0.2 (2)
C3—N1—C2—C1	−178.98 (18)	C7—N3—C6—C5	178.29 (19)
O2—C1—C2—C4	−173.6 (2)	O4—C5—C6—C8	175.7 (2)
O1—C1—C2—C4	6.0 (3)	O3—C5—C6—C8	−2.2 (3)
O2—C1—C2—N1	4.6 (3)	O4—C5—C6—N3	−1.8 (3)
O1—C1—C2—N1	−175.81 (19)	O3—C5—C6—N3	−179.68 (18)
C2—N1—C3—N2	0.6 (2)	C8—N4—C7—N3	0.5 (3)
C4—N2—C3—N1	−0.6 (2)	C6—N3—C7—N4	−0.5 (3)
N1—C2—C4—N2	−0.1 (2)	N3—C6—C8—N4	0.1 (2)
C1—C2—C4—N2	178.4 (2)	C5—C6—C8—N4	−177.7 (2)
C3—N2—C4—C2	0.4 (2)	C7—N4—C8—C6	−0.4 (3)

### (I) Bis(4-carboxy-1H-imidazol-3-ium) tetrachloridozincate monohydrate Hydrogen-bond geometry (Å, º)

**Table:**

*D*—H···*A*	*D*—H	H···*A*	*D*···*A*	*D*—H···*A*
O1*S*—H1*SA*···Cl2	0.83 (2)	2.42 (2)	3.192 (3)	155 (4)
O1*S*—H1*SB*···Cl4^i^	0.81 (2)	2.94 (4)	3.392 (2)	118 (3)
O1*S*—H1*SB*···Cl1^ii^	0.81 (2)	3.05 (4)	3.540 (3)	122 (4)
O1—H1*A*···Cl4^iii^	0.83 (2)	2.25 (2)	3.0723 (19)	171 (3)
O3—H3*A*···O1*S*^iv^	0.83 (2)	1.77 (2)	2.576 (3)	163 (3)
N1—H1*N*···Cl1	0.84 (2)	2.37 (2)	3.2105 (17)	178 (2)
N2—H2*N*···Cl1^i^	0.86 (2)	2.85 (2)	3.3787 (19)	122 (2)
N2—H2*N*···O2^i^	0.86 (2)	1.99 (2)	2.745 (2)	146 (2)
N3—H3*N*···Cl3^v^	0.83 (2)	2.37 (2)	3.1941 (18)	177 (2)
N4—H4*N*···Cl3^vi^	0.86 (2)	2.83 (2)	3.3586 (19)	121 (2)
N4—H4*N*···O4^i^	0.86 (2)	2.00 (2)	2.753 (2)	146 (2)

Symmetry codes: (i) *x*+1, *y*, *z*; (ii) *x*+1, *y*−1, *z*; (iii) −*x*, −*y*+1, −*z*+1; (iv) −*x*+1, −*y*, −*z*+1; (v) *x*, *y*, *z*+1; (vi) *x*+1, *y*, *z*+1.

### (II) bis(4-carboxy-1H-imidazol-3-ium) tetrachloridozincate bis(1H-imidazol-3-ium-4-carboxylato) monohydrate Crystal data

**Table:**

(C_4_H_4_N_2_O_2_)_2_[ZnCl_4_]·2C_4_H_5_N_2_O_2_H_2_O	*Z* = 2
*M**_r_* = 675.57	*F*(000) = 684
Triclinic, *P*1	*D*_x_ = 1.728 Mg m^−^^3^
*a* = 6.9369 (19) Å	Mo *K*α radiation, λ = 0.71073 Å
*b* = 6.9624 (15) Å	Cell parameters from 3388 reflections
*c* = 28.483 (8) Å	θ = 3.1–23.6°
α = 89.524 (9)°	µ = 1.42 mm^−^^1^
β = 85.622 (9)°	*T* = 200 K
γ = 71.202 (8)°	Block, clear colourless
*V* = 1298.3 (6) Å^3^	0.50 × 0.25 × 0.20 mm

### (II) bis(4-carboxy-1H-imidazol-3-ium) tetrachloridozincate bis(1H-imidazol-3-ium-4-carboxylato) monohydrate Data collection

**Table:**

Bruker SMART X2S benchtop diffractometer	4855 independent reflections
Radiation source: sealed microfocus tube	3619 reflections with *I* > 2σ(*I*)
Detector resolution: 8.3330 pixels mm^-1^	*R*_int_ = 0.042
ω scans	θ_max_ = 26.0°, θ_min_ = 2.9°
Absorption correction: multi-scan (SADABS; Bruker, 2013)	*h* = −8→8
*T*_min_ = 0.50, *T*_max_ = 0.76	*k* = −8→7
10560 measured reflections	*l* = −35→21

### (II) bis(4-carboxy-1H-imidazol-3-ium) tetrachloridozincate bis(1H-imidazol-3-ium-4-carboxylato) monohydrate Refinement

**Table:**

Refinement on *F*^2^	Primary atom site location: structure-invariant direct methods
Least-squares matrix: full	Secondary atom site location: difference Fourier map
*R*[*F*^2^ > 2σ(*F*^2^)] = 0.050	Hydrogen site location: mixed
*wR*(*F*^2^) = 0.135	H atoms treated by a mixture of independent and constrained refinement
*S* = 1.03	*w* = 1/[σ^2^(*F*_o_^2^) + (0.0739*P*)^2^ + 0.0203*P*] where *P* = (*F*_o_^2^ + 2*F*_c_^2^)/3
4855 reflections	(Δ/σ)_max_ < 0.001
395 parameters	Δρ_max_ = 0.63 e Å^−^^3^
16 restraints	Δρ_min_ = −0.71 e Å^−^^3^

### (II) bis(4-carboxy-1H-imidazol-3-ium) tetrachloridozincate bis(1H-imidazol-3-ium-4-carboxylato) monohydrate Special details

**Table:**

Geometry. All esds (except the esd in the dihedral angle between two l.s. planes) are estimated using the full covariance matrix. The cell esds are taken into account individually in the estimation of esds in distances, angles and torsion angles; correlations between esds in cell parameters are only used when they are defined by crystal symmetry. An approximate (isotropic) treatment of cell esds is used for estimating esds involving l.s. planes.

### (II) bis(4-carboxy-1H-imidazol-3-ium) tetrachloridozincate bis(1H-imidazol-3-ium-4-carboxylato) monohydrate Fractional atomic coordinates and isotropic or equivalent isotropic displacement parameters (Å^2^)

**Table:**

	*x*	*y*	*z*	*U*_iso_*/*U*_eq_	Occ. (<1)
Zn1	−0.05225 (7)	0.61778 (7)	0.24502 (2)	0.03731 (17)	
Cl1	−0.26017 (17)	0.82285 (19)	0.19411 (4)	0.0531 (3)	
Cl2	0.05785 (16)	0.29226 (16)	0.21504 (3)	0.0436 (3)	
Cl3	0.23787 (18)	0.6937 (2)	0.25097 (4)	0.0560 (3)	
Cl4	−0.22620 (16)	0.63506 (17)	0.31606 (3)	0.0408 (3)	
O1	0.3219 (4)	0.2856 (5)	0.02796 (9)	0.0471 (8)	
O2	0.1247 (4)	0.3358 (5)	0.09533 (10)	0.0517 (8)	
O3	0.3667 (5)	0.1705 (6)	0.39393 (12)	0.0661 (10)	
O4	0.4907 (5)	0.2123 (5)	0.46135 (10)	0.0559 (9)	
H4A	0.379 (5)	0.223 (8)	0.4788 (16)	0.084*	
O5	−0.1453 (5)	0.7598 (5)	0.50318 (10)	0.0537 (8)	
O6	−0.2533 (5)	0.6845 (6)	0.43625 (10)	0.0615 (9)	
O7	−0.2136 (5)	0.8281 (6)	0.07375 (11)	0.0685 (10)	
O8	−0.0196 (5)	0.7583 (5)	0.00627 (10)	0.0508 (8)	
H8A	−0.114 (6)	0.723 (8)	−0.0045 (16)	0.076*	
O1S	0.414 (2)	0.099 (2)	0.2899 (6)	0.070 (4)	0.60 (4)
H1A	0.334 (16)	0.176 (12)	0.272 (3)	0.105*	0.60 (4)
H1B	0.459 (16)	−0.021 (6)	0.279 (3)	0.105*	0.60 (4)
O2S	0.495 (5)	0.150 (4)	0.2703 (12)	0.093 (10)	0.40 (4)
H2A	0.381 (15)	0.15 (3)	0.262 (8)	0.14*	0.40 (4)
H2B	0.59 (2)	0.08 (3)	0.251 (5)	0.14*	0.40 (4)
N1	0.7400 (5)	0.3481 (6)	0.12509 (13)	0.0451 (9)	
H1N	0.870 (3)	0.342 (7)	0.1225 (15)	0.054*	
N2	0.4407 (5)	0.3466 (6)	0.14633 (11)	0.0392 (8)	
H2N	0.328 (4)	0.352 (6)	0.1626 (12)	0.047*	
N3	0.7314 (6)	0.1382 (5)	0.34378 (11)	0.0405 (8)	
H3N	0.644 (5)	0.134 (6)	0.3241 (12)	0.049*	
N4	1.0110 (6)	0.1551 (6)	0.36673 (14)	0.0460 (9)	
H4N	1.133 (4)	0.158 (7)	0.3638 (15)	0.055*	
N5	0.1092 (5)	0.6640 (5)	0.38684 (11)	0.0370 (8)	
H5N	0.024 (5)	0.646 (6)	0.3686 (12)	0.044*	
N6	0.3850 (5)	0.6882 (6)	0.41028 (12)	0.0437 (9)	
H6N	0.511 (4)	0.687 (7)	0.4100 (15)	0.052*	
N7	0.1081 (5)	0.8476 (5)	0.12179 (11)	0.0370 (8)	
H7N	0.006 (5)	0.834 (6)	0.1409 (12)	0.044*	
N8	0.4083 (5)	0.8406 (5)	0.09664 (12)	0.0392 (8)	
H8N	0.533 (4)	0.846 (6)	0.0945 (14)	0.047*	
C1	0.4601 (6)	0.3304 (6)	0.09817 (12)	0.0317 (9)	
C2	0.6497 (6)	0.3318 (6)	0.08465 (14)	0.0403 (10)	
H2	0.7095	0.3232	0.0533	0.048*	
C3	0.6112 (6)	0.3560 (7)	0.16165 (15)	0.0463 (11)	
H3	0.6366	0.3667	0.1937	0.056*	
C4	0.2872 (6)	0.3160 (6)	0.07221 (13)	0.0352 (9)	
C5	0.7016 (6)	0.1709 (6)	0.39137 (13)	0.0349 (9)	
C6	0.8780 (6)	0.1832 (6)	0.40570 (13)	0.0385 (9)	
H6	0.9044	0.207	0.437	0.046*	
C7	0.9189 (7)	0.1297 (6)	0.32923 (14)	0.0443 (10)	
H7	0.9775	0.1092	0.2977	0.053*	
C8	0.5038 (7)	0.1835 (6)	0.41683 (14)	0.0412 (10)	
C9	0.0765 (6)	0.6982 (6)	0.43471 (12)	0.0332 (9)	
C10	0.2521 (6)	0.7147 (6)	0.44949 (13)	0.0411 (10)	
H10	0.2774	0.7396	0.4808	0.049*	
C11	0.2965 (6)	0.6581 (7)	0.37284 (14)	0.0415 (10)	
H11	0.357	0.6361	0.3415	0.05*	
C12	−0.1239 (6)	0.7129 (6)	0.45951 (13)	0.0387 (9)	
C13	0.1228 (6)	0.8218 (6)	0.07366 (12)	0.0327 (9)	
C14	0.3108 (6)	0.8187 (6)	0.05808 (13)	0.0359 (9)	
H14	0.3661	0.804	0.0262	0.043*	
C15	0.2828 (6)	0.8583 (6)	0.13502 (13)	0.0395 (10)	
H15	0.313	0.8756	0.1664	0.047*	
C16	−0.0527 (6)	0.8025 (6)	0.04986 (13)	0.0371 (9)	

### (II) bis(4-carboxy-1H-imidazol-3-ium) tetrachloridozincate bis(1H-imidazol-3-ium-4-carboxylato) monohydrate Atomic displacement parameters (Å^2^)

**Table:**

	*U*^11^	*U*^22^	*U*^33^	*U*^12^	*U*^13^	*U*^23^
Zn1	0.0263 (3)	0.0572 (3)	0.0303 (2)	−0.0159 (2)	−0.00276 (18)	0.0010 (2)
Cl1	0.0298 (6)	0.0833 (8)	0.0449 (6)	−0.0162 (6)	−0.0066 (4)	0.0210 (5)
Cl2	0.0350 (6)	0.0569 (7)	0.0396 (5)	−0.0163 (5)	0.0002 (4)	−0.0053 (4)
Cl3	0.0384 (7)	0.0955 (9)	0.0465 (6)	−0.0383 (7)	−0.0061 (5)	0.0008 (6)
Cl4	0.0300 (6)	0.0627 (7)	0.0325 (5)	−0.0195 (5)	0.0005 (4)	−0.0008 (4)
O1	0.0278 (17)	0.083 (2)	0.0384 (15)	−0.0286 (17)	−0.0010 (12)	−0.0070 (14)
O2	0.0223 (16)	0.092 (2)	0.0451 (16)	−0.0247 (17)	0.0012 (12)	−0.0051 (15)
O3	0.034 (2)	0.106 (3)	0.067 (2)	−0.032 (2)	−0.0113 (16)	−0.0099 (19)
O4	0.0332 (19)	0.089 (2)	0.0449 (18)	−0.0209 (19)	0.0054 (13)	−0.0038 (16)
O5	0.0321 (18)	0.090 (2)	0.0411 (16)	−0.0239 (18)	0.0005 (13)	−0.0055 (15)
O6	0.0292 (18)	0.112 (3)	0.0530 (18)	−0.035 (2)	−0.0055 (14)	−0.0124 (18)
O7	0.0241 (18)	0.138 (3)	0.0505 (18)	−0.036 (2)	0.0012 (14)	−0.0083 (19)
O8	0.0326 (18)	0.086 (2)	0.0427 (16)	−0.0312 (18)	−0.0062 (13)	−0.0057 (15)
O1S	0.042 (6)	0.110 (6)	0.048 (6)	−0.008 (5)	−0.014 (5)	−0.020 (4)
O2S	0.060 (14)	0.130 (12)	0.068 (13)	0.008 (11)	−0.035 (11)	−0.019 (11)
N1	0.0172 (19)	0.063 (2)	0.060 (2)	−0.0188 (19)	−0.0057 (16)	−0.0067 (18)
N2	0.0176 (18)	0.060 (2)	0.0417 (18)	−0.0154 (18)	−0.0014 (14)	−0.0019 (16)
N3	0.032 (2)	0.052 (2)	0.0397 (18)	−0.0163 (18)	−0.0075 (15)	0.0016 (16)
N4	0.023 (2)	0.050 (2)	0.069 (2)	−0.0163 (18)	−0.0057 (17)	0.0118 (18)
N5	0.0210 (18)	0.052 (2)	0.0406 (18)	−0.0135 (17)	−0.0100 (14)	−0.0015 (15)
N6	0.0174 (18)	0.060 (2)	0.058 (2)	−0.0166 (18)	−0.0085 (16)	0.0067 (17)
N7	0.0236 (19)	0.056 (2)	0.0353 (17)	−0.0183 (17)	−0.0008 (13)	0.0015 (15)
N8	0.0171 (18)	0.050 (2)	0.056 (2)	−0.0168 (17)	−0.0054 (15)	0.0018 (16)
C1	0.020 (2)	0.040 (2)	0.0378 (19)	−0.0145 (18)	−0.0013 (15)	−0.0030 (16)
C2	0.020 (2)	0.055 (3)	0.044 (2)	−0.011 (2)	0.0009 (16)	−0.0067 (19)
C3	0.028 (2)	0.068 (3)	0.047 (2)	−0.020 (2)	−0.0079 (19)	−0.002 (2)
C4	0.023 (2)	0.045 (2)	0.041 (2)	−0.0149 (19)	−0.0041 (16)	0.0011 (17)
C5	0.031 (2)	0.036 (2)	0.040 (2)	−0.0118 (19)	−0.0084 (17)	0.0009 (17)
C6	0.036 (2)	0.043 (2)	0.040 (2)	−0.015 (2)	−0.0093 (18)	−0.0013 (17)
C7	0.039 (3)	0.050 (3)	0.044 (2)	−0.015 (2)	0.0016 (19)	0.0033 (19)
C8	0.027 (2)	0.049 (3)	0.050 (2)	−0.015 (2)	−0.0047 (18)	−0.0028 (19)
C9	0.020 (2)	0.044 (2)	0.0370 (19)	−0.0120 (19)	−0.0081 (15)	0.0046 (17)
C10	0.031 (2)	0.056 (3)	0.038 (2)	−0.014 (2)	−0.0110 (17)	−0.0017 (18)
C11	0.026 (2)	0.058 (3)	0.042 (2)	−0.015 (2)	−0.0014 (17)	0.0025 (19)
C12	0.025 (2)	0.052 (3)	0.041 (2)	−0.014 (2)	−0.0040 (17)	−0.0012 (18)
C13	0.022 (2)	0.044 (2)	0.0352 (19)	−0.0141 (19)	−0.0042 (15)	0.0015 (16)
C14	0.023 (2)	0.047 (2)	0.038 (2)	−0.013 (2)	−0.0007 (16)	−0.0019 (17)
C15	0.032 (2)	0.052 (3)	0.039 (2)	−0.017 (2)	−0.0118 (17)	0.0020 (18)
C16	0.020 (2)	0.055 (3)	0.039 (2)	−0.015 (2)	−0.0053 (16)	0.0019 (18)

### (II) bis(4-carboxy-1H-imidazol-3-ium) tetrachloridozincate bis(1H-imidazol-3-ium-4-carboxylato) monohydrate Geometric parameters (Å, º)

**Table:**

Zn1—Cl3	2.2577 (12)	N4—H4N	0.849 (19)
Zn1—Cl4	2.2589 (11)	N5—C11	1.317 (5)
Zn1—Cl1	2.2758 (12)	N5—C9	1.375 (5)
Zn1—Cl2	2.2948 (12)	N5—H5N	0.858 (19)
O1—C4	1.271 (4)	N6—C11	1.321 (5)
O2—C4	1.229 (4)	N6—C10	1.367 (5)
O3—C8	1.220 (5)	N6—H6N	0.868 (19)
O4—C8	1.277 (5)	N7—C15	1.321 (5)
O4—H4A	0.87 (2)	N7—C13	1.376 (4)
O5—C12	1.274 (5)	N7—H7N	0.889 (19)
O6—C12	1.222 (5)	N8—C15	1.325 (5)
O7—C16	1.224 (5)	N8—C14	1.367 (5)
O8—C16	1.267 (5)	N8—H8N	0.872 (19)
O8—H8A	0.851 (19)	C1—C2	1.345 (5)
O1S—H1A	0.85 (2)	C1—C4	1.487 (5)
O1S—H1B	0.84 (2)	C2—H2	0.95
O2S—H2A	0.85 (2)	C3—H3	0.95
O2S—H2B	0.85 (2)	C5—C6	1.347 (5)
N1—C3	1.309 (5)	C5—C8	1.479 (5)
N1—C2	1.375 (5)	C6—H6	0.95
N1—H1N	0.884 (19)	C7—H7	0.95
N2—C3	1.312 (5)	C9—C10	1.357 (5)
N2—C1	1.370 (5)	C9—C12	1.484 (5)
N2—H2N	0.871 (19)	C10—H10	0.95
N3—C7	1.318 (5)	C11—H11	0.95
N3—C5	1.365 (5)	C13—C14	1.339 (5)
N3—H3N	0.866 (19)	C13—C16	1.481 (5)
N4—C7	1.327 (6)	C14—H14	0.95
N4—C6	1.361 (5)	C15—H15	0.95
			
Cl3—Zn1—Cl4	111.21 (4)	N1—C3—H3	126.0
Cl3—Zn1—Cl1	112.42 (5)	N2—C3—H3	126.0
Cl4—Zn1—Cl1	109.31 (5)	O2—C4—O1	126.3 (4)
Cl3—Zn1—Cl2	104.23 (5)	O2—C4—C1	117.2 (3)
Cl4—Zn1—Cl2	110.95 (4)	O1—C4—C1	116.5 (3)
Cl1—Zn1—Cl2	108.62 (5)	C6—C5—N3	106.6 (3)
C8—O4—H4A	122 (4)	C6—C5—C8	132.8 (4)
C16—O8—H8A	113 (3)	N3—C5—C8	120.6 (3)
H1A—O1S—H1B	111 (5)	C5—C6—N4	106.8 (3)
H2A—O2S—H2B	113 (6)	C5—C6—H6	126.6
C3—N1—C2	109.4 (3)	N4—C6—H6	126.6
C3—N1—H1N	132 (3)	N3—C7—N4	107.5 (4)
C2—N1—H1N	118 (3)	N3—C7—H7	126.3
C3—N2—C1	109.7 (3)	N4—C7—H7	126.3
C3—N2—H2N	128 (3)	O3—C8—O4	125.6 (4)
C1—N2—H2N	122 (3)	O3—C8—C5	118.2 (4)
C7—N3—C5	109.6 (3)	O4—C8—C5	116.2 (4)
C7—N3—H3N	121 (3)	C10—C9—N5	106.6 (3)
C5—N3—H3N	129 (3)	C10—C9—C12	133.0 (3)
C7—N4—C6	109.4 (4)	N5—C9—C12	120.4 (3)
C7—N4—H4N	120 (3)	C9—C10—N6	106.2 (3)
C6—N4—H4N	130 (3)	C9—C10—H10	126.9
C11—N5—C9	109.5 (3)	N6—C10—H10	126.9
C11—N5—H5N	124 (3)	N5—C11—N6	107.7 (3)
C9—N5—H5N	126 (3)	N5—C11—H11	126.1
C11—N6—C10	110.0 (3)	N6—C11—H11	126.1
C11—N6—H6N	125 (3)	O6—C12—O5	126.3 (4)
C10—N6—H6N	125 (3)	O6—C12—C9	117.6 (3)
C15—N7—C13	109.3 (3)	O5—C12—C9	116.1 (3)
C15—N7—H7N	126 (3)	C14—C13—N7	106.7 (3)
C13—N7—H7N	124 (3)	C14—C13—C16	133.2 (3)
C15—N8—C14	109.4 (3)	N7—C13—C16	120.1 (3)
C15—N8—H8N	128 (3)	C13—C14—N8	107.0 (3)
C14—N8—H8N	122 (3)	C13—C14—H14	126.5
C2—C1—N2	106.3 (3)	N8—C14—H14	126.5
C2—C1—C4	133.6 (3)	N7—C15—N8	107.6 (3)
N2—C1—C4	120.2 (3)	N7—C15—H15	126.2
C1—C2—N1	106.6 (3)	N8—C15—H15	126.2
C1—C2—H2	126.7	O7—C16—O8	126.3 (4)
N1—C2—H2	126.7	O7—C16—C13	117.8 (3)
N1—C3—N2	108.0 (4)	O8—C16—C13	115.9 (3)
			
C3—N2—C1—C2	−0.4 (5)	C11—N5—C9—C10	0.5 (5)
C3—N2—C1—C4	179.6 (4)	C11—N5—C9—C12	179.7 (4)
N2—C1—C2—N1	0.2 (5)	N5—C9—C10—N6	−0.5 (5)
C4—C1—C2—N1	−179.9 (4)	C12—C9—C10—N6	−179.6 (4)
C3—N1—C2—C1	0.1 (5)	C11—N6—C10—C9	0.4 (5)
C2—N1—C3—N2	−0.4 (5)	C9—N5—C11—N6	−0.2 (5)
C1—N2—C3—N1	0.5 (5)	C10—N6—C11—N5	−0.1 (5)
C2—C1—C4—O2	−174.2 (5)	C10—C9—C12—O6	−177.8 (5)
N2—C1—C4—O2	5.7 (6)	N5—C9—C12—O6	3.2 (6)
C2—C1—C4—O1	5.4 (7)	C10—C9—C12—O5	4.0 (7)
N2—C1—C4—O1	−174.7 (4)	N5—C9—C12—O5	−175.0 (4)
C7—N3—C5—C6	0.3 (5)	C15—N7—C13—C14	−0.4 (5)
C7—N3—C5—C8	−179.0 (4)	C15—N7—C13—C16	179.2 (4)
N3—C5—C6—N4	−0.8 (5)	N7—C13—C14—N8	0.5 (5)
C8—C5—C6—N4	178.4 (4)	C16—C13—C14—N8	−179.0 (4)
C7—N4—C6—C5	1.1 (5)	C15—N8—C14—C13	−0.4 (5)
C5—N3—C7—N4	0.4 (5)	C13—N7—C15—N8	0.1 (5)
C6—N4—C7—N3	−0.9 (5)	C14—N8—C15—N7	0.2 (5)
C6—C5—C8—O3	178.6 (5)	C14—C13—C16—O7	−174.2 (5)
N3—C5—C8—O3	−2.3 (6)	N7—C13—C16—O7	6.3 (6)
C6—C5—C8—O4	0.2 (7)	C14—C13—C16—O8	6.5 (7)
N3—C5—C8—O4	179.3 (4)	N7—C13—C16—O8	−173.0 (4)

### (II) bis(4-carboxy-1H-imidazol-3-ium) tetrachloridozincate bis(1H-imidazol-3-ium-4-carboxylato) monohydrate Hydrogen-bond geometry (Å, º)

**Table:**

*D*—H···*A*	*D*—H	H···*A*	*D*···*A*	*D*—H···*A*
O4—H4*A*···O5^i^	0.87 (2)	1.63 (2)	2.476 (4)	163 (5)
O4—H4*A*···O6^i^	0.87 (2)	2.52 (4)	3.205 (4)	136 (4)
O8—H8*A*···O1^ii^	0.85 (2)	1.65 (2)	2.484 (4)	166 (5)
O8—H8*A*···O2^ii^	0.85 (2)	2.63 (4)	3.162 (4)	122 (4)
O1*S*—H1*A*···Cl2	0.85 (2)	2.52 (5)	3.330 (9)	159 (10)
O1*S*—H1*B*···Cl1^iii^	0.84 (2)	2.97 (9)	3.546 (15)	128 (10)
O1*S*—H1*B*···Cl4^iii^	0.84 (2)	2.93 (7)	3.499 (11)	127 (8)
O2*S*—H2*A*···Cl2	0.85 (2)	2.61 (12)	3.382 (17)	150.20
O2*S*—H2*B*···Cl1^iii^	0.85 (2)	2.36 (10)	3.13 (2)	150 (17)
N1—H1*N*···O2^iv^	0.88 (2)	1.86 (2)	2.712 (4)	160 (4)
N2—H2*N*···Cl2	0.87 (2)	2.44 (2)	3.297 (3)	167 (4)
N3—H3*N*···O1*S*	0.87 (2)	2.01 (2)	2.860 (10)	168 (4)
N3—H3*N*···O2*S*	0.87 (2)	1.89 (2)	2.739 (12)	165 (4)
N4—H4*N*···O3^iv^	0.85 (2)	1.92 (3)	2.677 (5)	148 (4)
N5—H5*N*···Cl4	0.86 (2)	2.39 (2)	3.247 (3)	173 (4)
N6—H6*N*···O6^iv^	0.87 (2)	1.85 (3)	2.661 (4)	156 (4)
N7—H7*N*···Cl1	0.89 (2)	2.32 (2)	3.205 (3)	175 (4)
N8—H8*N*···O7^iv^	0.87 (2)	1.78 (2)	2.628 (4)	164 (4)
C2—H2···O8^v^	0.95	2.55	3.414 (5)	152
C3—H3···Cl2^iv^	0.95	2.91	3.454 (4)	118
C6—H6···O5^vi^	0.95	2.54	3.400 (5)	151
C7—H7···Cl2^iv^	0.95	2.77	3.599 (4)	146
C10—H10···O4^vi^	0.95	2.49	3.345 (5)	151
C11—H11···Cl3	0.95	2.75	3.523 (4)	139
C11—H11···Cl4^iv^	0.95	2.92	3.528 (4)	123
C14—H14···O1^v^	0.95	2.47	3.303 (5)	147
C15—H15···Cl3	0.95	2.8	3.511 (4)	132

Symmetry codes: (i) −*x*, −*y*+1, −*z*+1; (ii) −*x*, −*y*+1, −*z*; (iii) *x*+1, *y*−1, *z*; (iv) *x*+1, *y*, *z*; (v) −*x*+1, −*y*+1, −*z*; (vi) −*x*+1, −*y*+1, −*z*+1.
